# Exploration and optimization of different charge transport layers for Cs_4_CuSb_2_Cl_12_ based perovskite solar cells

**DOI:** 10.1038/s41598-025-10731-6

**Published:** 2025-07-11

**Authors:** Kazi Md Sadat, M. Khalid Hossain, M. Shihab Uddin, P. Prabhu, Ankita Aggarwal, K. Gopalakrishna, P. Sasi Kiran, Alok Kumar Mishra, Sanjeev Kumar Shah, Sahjahan Islam, Abdullah M. S. Alhuthali, Magda H. Abdellattif, V. K. Mishra

**Affiliations:** 1 Dept. of Electrical and Electronic Engineering, Mymensingh Engineering College, Mymensingh, 2200 Bangladesh; 2 Institute of Electronics, Atomic Energy Research Establishment, Bangladesh Atomic Energy Commission, Dhaka, 1349 Bangladesh; 3 Department of Advanced Energy Engineering Science, Interdisciplinary Graduate School of Engineering Sciences, Kyushu University, Fukuoka, 816-8580 Japan; 4 Department of Computer Science and Engineering, Daffodil International University, Dhaka, 1216 Bangladesh; 5 Research and Innovation Cell, Rayat Bahra University, Mohali, Punjab India; 6 Department of Mechanical Engineering, Mattu University, Mettu, 318 Ethiopia; 7 Department of ECE, Chandigarh Engineering College, Chandigarh Group of Colleges-Jhanjeri, Mohali, Punjab 140307 India; 8 Department of Electronics and Communication Engineering, School of Engineering and Technology, JAIN (Deemed to be University), Bangalore, Karnataka India; 9 Department of EEE, Raghu Engineering College, Visakhapatnam, 531162 Andhra Pradesh India; 10 Department of Electrical & Electronics Engineering, Siksha ’O’ Anusandhan (Deemed to be University), Bhubaneswar, 751030 Odisha India; 11 Department of Electronics & Communication engineering, Uttaranchal Institute of Technology, Uttaranchal University, Dehradun, 248007 Uttarakhand India; 12 Department of Physics & Astronomy, East Texas A&M University, Commerce, TX 75428 USA; 13 Department of Physics, College of Sciences, Taif University, P.O. Box 11099, Taif, 21944 Saudi Arabia; 14 Department of Chemistry, College of Science, University College of Taraba, Taif University, P.O. Box 11099, Taif, Saudi Arabia; 15 School of Chemical Engineering, Yeungnam University, Gyeongsan, 38541 Republic of Korea

**Keywords:** Perovskite solar cell, Cs_4_CuSb_2_Cl_12_ absorber, ZnSe ETL, MWCNT HTL, SCAPS-1D, Materials science, Physics

## Abstract

Recently, lead-free Cs_4_CuSb_2_Cl_12_ has garnered attention as an excellent material to be used as an absorber of perovskite solar cells (PSCs). In this work, Cs_4_CuSb_2_Cl_12_ absorber-based PSCs were studied and the conditions to get high performance for PSCs were investigated. Here, six different materials for electron transport layers (ETLs) and 10 different materials for hole transport layers (HTLs) were studied. A numerical approach was followed by using SCAPS-1D simulator. During the work, various device parameters of PSC were investigated such as thickness variation of the absorber and ETL layers, acceptor density variation of the absorber and HTL layers, variation of the donor density of the ETL layer, and effect of total defect density of absorber. Also, other parameters such as the impact of resistance, temperature, J-V graph, Q-E graph, and carrier generation rate at different positions of the PSCs were assessed. Among the studied 10 HTL materials, MWCNTs outperformed other studied materials, hence it was selected for further investigations. Then the structures were optimized based on the device parameters outcome, and the structure having MZO and STO ETLs both showed the maximum power conversion efficiency (PCE) of 28.23%. (Al/FTO/MZO/Cs_4_CuSb_2_Cl_12_/MWCNTs/Au) the structure showed an open-circuit voltage (V_oc_) of 1.249 V, short-circuit current density (J_sc_) of 25.11 mA/cm^2^ and a fill factor (FF) of 90.1%. The performance was also evaluated with respect to key electrical parameters. Optimum performance was achieved at a series resistance of 1 Ω·cm² and a shunt resistance of 1000 Ω·cm², beyond which performance gains saturated. The other best-performing STO ETL-based (Al/FTO/STO/Cs_4_CuSb_2_Cl_12_/MWCNTs/Au) structure had V_oc_ of 1.25 V, J_sc_ of 25.11 mA/cm^2^, and FF of 90.01% Under the optimized condition other structure with CdS, PC_61_BM, SnS_2_ and ZnSe ETLs showed PCE of 27.68%, 27.8%, 25.67% and 28.22%. This work gives good insights into several Cs_4_CuSb_2_Cl_12_ based PSC structures and shows in the future they have great potential to be developed practically for highly efficient performances.

## Introduction

To overcome the global challenge of heavy reliance on natural resources for energy and to ensure the smooth transition in the future to green energy the policy makers are now focusing on the development of renewable energy sources^1–6^. Different renewable sources of energy such as windmills, hydroelectricity, bioenergy, and solar cells^7–12^ are the most reliable and have the quality to be used on a large scale^13–16^. Among these options, solar cells have the best potential to develop, can be used everywhere from a smaller scale to a larger scale, have good durability, and need less effort to maintain making them the best energy source for usage^17–19^. Solar cells convert the energy of photons and convert them to generate electricity. It is a clean and reliable source of energy. There are different types of solar cell technologies: DSCC^20–23^crystalline solar cells^24^polycrystalline solar cells^25^silicon solar cells^26–29^and thin-film solar cells (TFSC)^17,30,31^. TFSC needs comparatively less material than other technologies and it is more environment friendly. Among the several types of TFSC perovskite solar cells^32–39^one of the best-performing technologies shows high power conversion efficiency (PCE)^40–43^. It is cheap, environment-friendly, and suitable for production in industries^44,45^. It shows convenient electrical, and optical properties and a good absorption coefficient making it an excellent choice for the usage of solar cells^46,47^.

Copper-Zinc-Telluride-Sulfide (CZTS)^48^Copper-Zinc-Telluride selenide (CZTSe)^49,50^CdTe^11^and etc. inorganic absorber material-based PSC currently popular in the scientific studies for their potentials. Recently, several organic-inorganic absorber materials have gained a lot of attraction in PSC research. Formamidinium lead Iodide (FAPbI_3_)^51^Formamidinium Tin Iodide (FASnI_3_)^52^Methylammonium Tin Iodide (MASnI_3_)^53^Methylammonium Lead Iodide (MAPbI_3_)^54^ are some of the bests performing organic-inorganic absorbers of TFSC. Many perovskites have AMX_3_ general formula where A can be Cesium (Cs), Flouridinium (FA), or methyl ammonium (MA); X represents halogen such as Cl, Br, I and M denotes cations such as Pb, Sn, or Ge based organic-inorganic have been discovered. These perovskites show remarkable optoelectronic properties such as a high absorption coefficient of > 10^5^ cm^− 1^, tunable band gap, and a wide range of light for absorption^9,10^. Having these suitable properties perovskites with AMX_3_ general formula can be used for optoelectronic devices including LEDs^50,55,56^photodetectors^57^and solar cells^58,59^. However, the presence of Pb in perovskites makes them detrimental to the environment^60^ so choosing Pb for solar cells is not ideal. One of the solutions is to replace Pb^2+^ with Sn^2+^ and Ge^2+^ which is not viable since both make perovskite centers susceptible to oxidation, thus hampering the longevity of perovskites^61^. So, heterovalent Bi^3+^ and Sb^3+^ have been used as lead-free alternatives in zero-dimensional and two-dimensional chalcogenides^62,63^. Lately, a new three-dimensional A_2_B’B” X_6_ double perovskite structure has been studied^64,65^. This structure can contain non-toxic cations and metal ions and shows stability. However, Cs_2_AgBiBr_6_, which shows the best optoelectronic properties among the structures without Pb presence that follows the AMX_3_ general formula, has a wide bandgap of 2.19 eV that limits the potential to be used in solar cells^66,67^. However, by applying techniques such as pressure-assisted band gap tuning^68^ and dilute alloying^69^ the bandgap can be reduced to make it applicable in photovoltaics.

Mostly, 3D structured double perovskites which are lead-free are bad absorbers due to the property of large indirect bandgap. After exploring suitable perovskites, Cs_4_CuSb_2_Cl_12_ (CCSC) showed suitable photoelectric properties having a band gap of 1eV^69^. Cs_4_CuSb_2_Cl_12_ bulk has high electron effective mass which causes slow electron mobility and thus deteriorates performances of solar cells. The strategy to decrease the particle size of Cs_4_CuSb_2_Cl_12_ (CCSC) to nanoscale paved the way to tune energy band structure^69^. In two experimental studies, the average particle size of (CCSC) with nanocrystals (NCs) was constructed as 3 nm^70^ and 3.9 nm^71^ using different research methods. Perovskites with nanocrystals have some salient properties including variable bandgap, high electrical conductivity, and large absorption spectrum which are conducive to the applications of photovoltaics^72–74^.

Exploration on Cs_4_CuSb_2_Cl_12_ absorber-based PSCs structure designing is in the initial stages since the material is developed in the recently made suitable for application. In one study, 16.6% PCE^75^ was obtained using these Cs_4_CuSb_2_Cl_12_ NCs. In a simulation study where (FTO/TiO_2_/CCSCNCs/Cu_2_O/Au) PSC device structure was studied and showed a PCE of 23.07%^76^ when optimized using the SCAPS-1D simulator. (FTO/WS_2_/Cs_4_CuSb_2_Cl_12_/CuSbS_2_/Ni) PSC device structure was proposed after examining several materials for ETL and HTL layers in one of the recent works on Cs_4_CuSb_2_Cl_12_ absorber PSCs. 23.10% PCE^77^ was achieved after the optimization of the PSC device using SCAPS-1D simulator. In another research article, after examining 244 different PSC device structures using SCAPS-1D simulator, two different device structures were proposed, one structure showed 29.71% PCE having an HTL layer, and the other device structure was designed without any HTL layer but Pt as the back contact showed 29.61% PCE^78^. In both device structures, SnO_2_ was used as an ETL layer.

There is a big gap in research studies on the ETLs and HTLs for Cs_4_CuSb_2_Cl_12_ PSCs. Understanding the potential of this absorber and limited conducted studies on ETL and HTL materials, here six ETL materials were investigated: PC_61_BM, ZnSe, STO, MZO, CdS, and SnS_2_. Besides 10 HTLs were also studied as follows: ZnTe, Sb_2_S_3_, MWCNTs, MoO_3_, Cu: NiO, MASnBr_3_, TiO_2_:N, CZGS, NiCo_2_O_4_ and CuAlO_2_. A broad exploration of the performances of the PSC devices for different charge transport layer materials was conducted. This study examines designs of many possible device structures of the Cs_4_CuSb_2_Cl_12−_PSC technology which could give useful insights in the future for practical implementations. The objectives of this paper could be summarized as: (i) exploration of ETL and HTL materials for the Cs_4_CuSb_2_Cl_12_-absorber-based PSC technology, (ii) study different PSC structures and optimize them to get highly efficient device performances, (iii) assess the performances of the devices for optimum conditions, (iv) propose the best device structures after evaluating practical challenges, and (v) provide an outline of device structure modifications for future works.

## Methodology

### SCAPS-1D simulation

Here, the SCAPS-1D tool was used which was developed by the Electrical and Information Engineering Department of the University of Gent of Belgium^59,79–82^. This tool is specifically designed for the simulation works of solar cells which has been proved reliable when compared to the experimental research in many works. This tool is useful in solar cell research works since close results of real performances can be replicated in simulation and it is now widely used. SCAPS-1D tool is effective in TFSC simulation works. It can be used to study performance characteristics such as J_sc_, V_oc_, FF, and PCE of solar cells. Besides, it can be used to study different graphs such as the J-V curve, Q-V curve, C-f curve, band diagram, etc. Other factors that impact TFSCs such as series resistance, shunt resistance, temperature, etc. can studied. The device structure of PSCs can design and parameters of materials such as band diagram, mobility of electrons and holes, electron affinity, doping density of carriers, several types of defects such as defects at the interfaces of material layers, recombination defect, etc. can be defined in the device’s structure designing. Up to seven different layers of materials can be used to construct the expected device structures. SCAPS-1D uses several equations to calculate the performances of PSCs such as Poisson’s equation, electron continuity equation, hole continuity equation, drift, and diffusion of carriers’ equations, etc. This tool uses numerical methods such as Newton-Raphson, continuity equations to solve equations.

The Poisson’s equation can be defined as Eq. 1. Here, ψ is represented as the potential of the electric field, ∈₀ is denoted as permittivity in free space whereas ∈_r_ is represented as permittivity in a relative medium. q is denoted as the amount of charge, ρ_n_ is represented as the distribution of electrons, and ρ_p_ is represented as the distribution of holes. N_A_ is the density of acceptor carriers and N_D_ is symbolized as the density of donor carriers.

1 $$\frac{{d^{2} }}{{dx^{2} }}\user2{\psi }\left( x \right) = \frac{q}{{\varepsilon _{o} \varepsilon _{r} }}\left[ {p\left( x \right) - n\left( x \right) - N_{D} - N_{A} + \rho _{p} - \rho _{n} } \right]$$

The continuity equations can be defined as Eqs. 2 and 3. Here, Eq. 2 is the continuity equation of electrons, and Eq. 3 is the continuity equation of holes. Here, J_n_ is denoted as the current density for electrons and J_p_ is denoted as the current densities of holes. G_n_ is denoted as the electron recombination rate and G_p_ is symbolized as the hole recombination rate.

2 $$\:-\frac{\partial\:{J}_{n}}{\partial\:\text{x}}-{U}_{n}+G=\frac{{\partial\:}_{n}}{\partial\:\text{t}}$$

3 $$\:-\frac{\partial\:{J}_{p}}{\partial\:\text{x}}-{U}_{p}+G=\frac{{\partial\:}_{p}}{\partial\:\text{t}}$$

Equation 4 and Eq. 5 represent the relation of charge carrier drift-diffusions to calculate the current densities of the electrons and holes of solar cells. Here q represents the total number of charges; µ_n_ and µ_p_ are the mobility of electron and hole carriers respectively.

4 $$\:{J}_{n}=\:-\frac{{\:n\mu\:}_{n\:}}{q}\frac{{\partial\:{E}_{Fn}}_{\:}}{\partial\:\:x}$$

5 $$\:{J}_{p}=\:-\frac{{\:n\mu\:}_{p\:}}{q}\frac{{\partial\:{E}_{Fp}}_{\:}}{\partial\:\:x}$$

The performance of a photovoltaic cell is measured by the quality fill factor (FF). FF depends on the product value of the maximum voltage and maximum current representing the maximum power to the calculated theoretical power (Pt), considering the open circuit voltage (V_oc_) and short circuit current (J_sc_) shown in Eq. 6. The power conversion efficiency (PCE) (Eq. 7) shows the ratio of output energy that can be found from the photovoltaic solar cells to the given input energy. Its performance relies on the product of V_oc_, J_sc_, and FF to the given input power as shown in the Eq. 

6 $$\:{FF}=\:-\frac{{\:P}_{max\:}}{{\:P}_{t\:}}=\frac{{\:{\:V}_{max\:}I}_{max\:\:}}{{\:{\:V}_{oc\:}J}_{sc\:\:}}$$

7 $$\:PCE=\frac{{\:{\:V}_{oc}{{\:J}_{sc\:}FF}_{\:\:}}_{\:\:\:\:\:}}{{\:P}_{in\:}}$$

### Structure of the Cs_4_CuSb_2_Cl_12_ -absorber devices

**Fig. 1:**
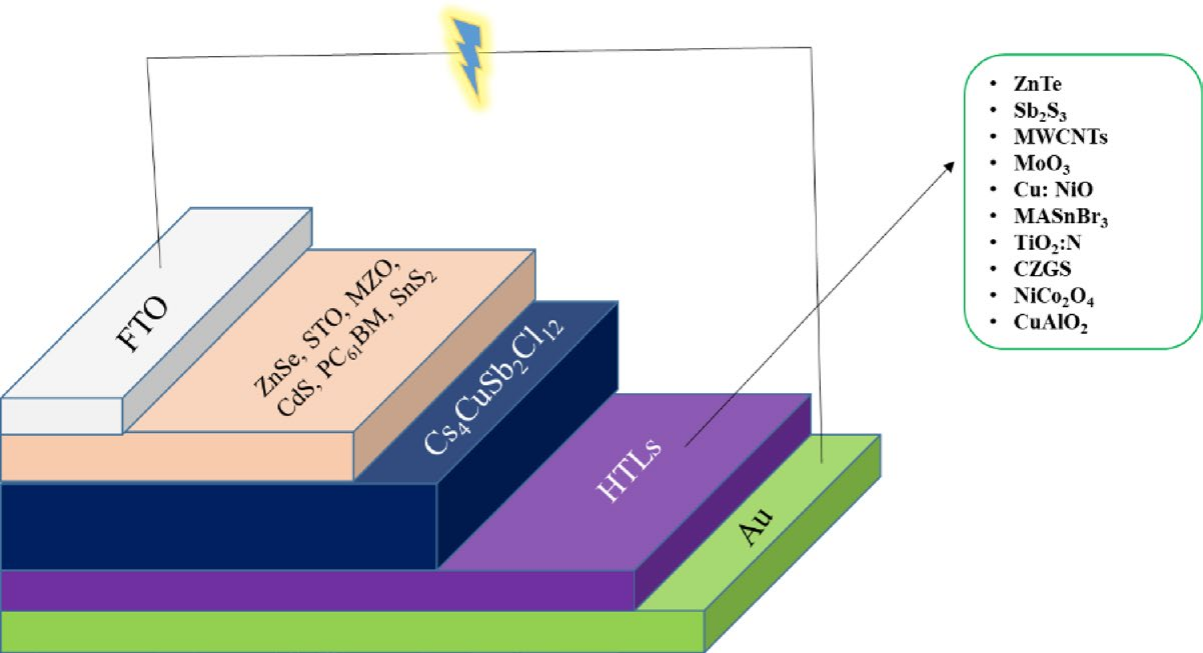
Cs_4_CuSb_2_Cl_12_ based proposed PSC device structures.

In this work, Al/FTO/ETL/ Cs_4_CuSb_2_Cl_12_/HTL/Au structure devices were studied. Six different ETLs were studied as follows: CdS, PC_61_BM, SnS_2_, MZO, STO, and ZnSe. Besides, 10 HTLs were investigated for the Cs_4_CuSb_2_Cl_12_ absorber-based structure. Sb_2_Se_3_, Cu doped with NiO. Cu_2_Te, ZnTe, MoTe_2_, CuAlO_2_, CZGS, MASnBr_3_, MoO_3_, MWCNTs, NiCo_2_O_4_, and TiO_2_ doped with nitrogen were initially chosen for device structures to study. Here, gold (Au) was selected as the back contact^50^ and Al as the front contact layer. FTO was chosen as the window layer for the structure devices. This work, extensively focused on studying the performances of ETL and HTL layers. All the configurations of the device structure were investigated using the SCAPS-1D tool (input parameters are given in Tables 1, 2 and 3) under the 1.5 AM solar radiation condition and 100 mW/cm^2^ power density was applied. The Cs4CuSb2Cl12-based proposed PSC device structures are shown in Fig. 1.

**Table 1:** Input parameters of interface defect layers^83^.

Interface	Defect type	Capture cross section: electrons/holes (cm^2^)	Energetic distribution	Reference for defect energy level	Total density (cm^− 2^) (integrated over all energies)
ETL/Cs_4_CuSb_2_Cl_12_	Neutral	1.0 × 10^− 17^ 1.0 × 10^− 18^	Single	Above the VB maximum	1.0 × 10^10^
Cs_4_CuSb_2_Cl_12_/HTL	Neutral	1.0 × 10^− 18^ 1.0 × 10^− 19^	Single	Above the VB maximum	1.0 × 10^10^

**Table 2:** Material input parameters for FTO, absorber (Cs₄CuSb₂Cl₁₂), and different ETL layers in the simulated PSC structures.

Material property	FTO	Cs_4_CuSb_2_Cl_12_	PC_61_BM	ZnSe	STO	MZO	CdS	SnS2
Thickness (nm)	200	700	25	70	70	150	50	150
Eg (eV)	3.5	1.6	2.1	2.81	3.2	3.3	2.4	1.85
Χ (eV)	4.00	3.74	4	4.09	4	4	4.18	4.26
εr	9.00	10	18	8.6	8.7	66	10	17.7
N_C_ (1/cm^3^)	2.2$\:\times\:$10^18^	4.5$\:\times\:$10^19^	2.2$\:\times\:$10^18^	2.2$\:\times\:$10^18^	1.7$\:\times\:$10^19^	2.2$\:\times\:$10^18^	2.2$\:\times\:$10^18^	7.32$\:\times\:$10^18^
N_V_ (1/cm^3^)	1.8$\:\times\:$10^19^	1.6$\:\times\:$10^19^	1.8$\:\times\:$10^19^	1.8$\:\times\:$10^18^	2$\:\times\:$10^20^	1.8$\:\times\:$10^19^	1.9$\:\times\:$10^19^	1$\:\times\:$10^19^
V_thn_, (cm s^− 1^)	10^7^	10^7^	10^7^	10^7^	10^7^	10^7^	10^7^	10^7^
V_thp_,(cm s^− 1^)	10^7^	10^7^	10^7^	10^7^	10^7^	10^7^	10^7^	10^7^
µ_n_, (cm^2^/Vs)	20	2.5	2$\:\times\:$10^−3^	4$\:\times\:$10^2^	5.3$\:\times\:$10^3^	100	100	50
µ_h_ (cm^2^/Vs)	10	2.5	2$\:\times\:$10^−3^	1.1$\:\times\:$10^2^	6.6$\:\times\:$10^2^	25	25	25
N_D_ (1/cm3)	10^18^	0	1$\:\times\:$10^17^	1$\:\times\:$10^18^	2$\:\times\:$10^16^	1$\:\times\:$10^18^	1$\:\times\:$10^18^	9.85$\:\times\:$10^19^
N_A_ (1/cm^3^)	0	10^13^	0	0	0	0	0	0
Total density (cm^− 3^)	10^15^	1$\:\times\:$10^13^	2$\:\times\:$10^15^	1$\:\times\:$10^15^	10^15^	10^15^	1$\:\times\:$10^15^	10^14^
References		^84,85^	^86^	^87^	^87^	^88^	^89^	^88^

**Table 3:** Material input parameters for various HTLs are considered in the simulation study.

Material property	ZnTe	Sb_2_S_3_	MWCNTs	MoO_3_	Cu: NiO	MASnBr_3_	TiO_2_:*N*	CZGS	NiCo_2_O_4_	CuAlO_2_
Thickness (nm)	250	250	600	100	100	200	50	200	70	350
Eg (eV)	2.25	1.62	1.55	3.0	3.55	2.15	3	1.95	2.3	3.46
Χ (eV)	3.73	3.7	3.64	2.3	1.8	3.39	2.2	3.67	3.48	2.5
εr	7.3	7.08	9	18	11.75	8.2	3	10	11.9	60
N_C_ (1/cm^3^)	2.2$\:\times\:$10^18^	20$\:\times\:$10^18^	2.2$\:\times\:$10^18^	1$\:\times\:$10^19^	2.2 × 10^21^	1$\:\times\:$10^20^	1.3$\:\times\:$10^14^	2.2$\:\times\:$10^18^	2.2$\:\times\:$10^18^	2.2$\:\times\:$10^18^
N_V_ (1/cm^3^)	1.8$\:\times\:$10^19^	10$\:\times\:$10^18^	1.8$\:\times\:$10^19^	2.2$\:\times\:$10^18^	1.8$\:\times\:$10^19^	1$\:\times\:$10^18^	1.3$\:\times\:$10^15^	1.8$\:\times\:$10^19^	1$\:\times\:$10^19^	1.8$\:\times\:$10^19^
V_thn_, (cm s^− 1^)	10^7^	10^7^	10^7^	10^7^	10^7^	10^7^	10^7^	10^7^	10^7^	10^7^
V_thp_,(cm s^− 1^)	10^7^	10^7^	10^7^	10^7^	10^7^	10^7^	10^7^	10^7^	10^7^	10^7^
µ_n_, (cm^2^/Vs)	300	9.8	100	210	1.5$\:\times\:$10^−2^	1.6	2	60	1.05	2
µ_h_ (cm^2^/Vs)	100	10	38.69	210	1.5$\:\times\:$10^−2^	1.6	2	20	1.61	8.6
N_D_ (1/cm3)			0	0	0	0	0	10	0	0
N_A_ (1/cm^3^)	2.0$\:\times\:$10^18^	5.7$\:\times\:$10^15^	4$\:\times\:$10^18^	1$\:\times\:$10^18^	1$\:\times\:$10^18^	1$\:\times\:$10^18^	1.3$\:\times\:$10^14^	2$\:\times\:$10^16^	1$\:\times\:$10^18^	3$\:\times\:$10^18^
Total density (cm^− 3^)	1$\:\times\:$10^14^	1$\:\times\:$10^14^	8$\:\times\:$10^14^	1$\:\times\:$10^15^	1$\:\times\:$10^16^	1$\:\times\:$10^15^	1$\:\times\:$10^15^	1$\:\times\:$10^15^	1$\:\times\:$10^15^	1$\:\times\:$10^15^
References	^90^	^91^	^92^	^93^	^94^	^95^	^96^	^97^	^98^	^87^

## Result and discussion

### Energy band alignment

**Fig. 2:**
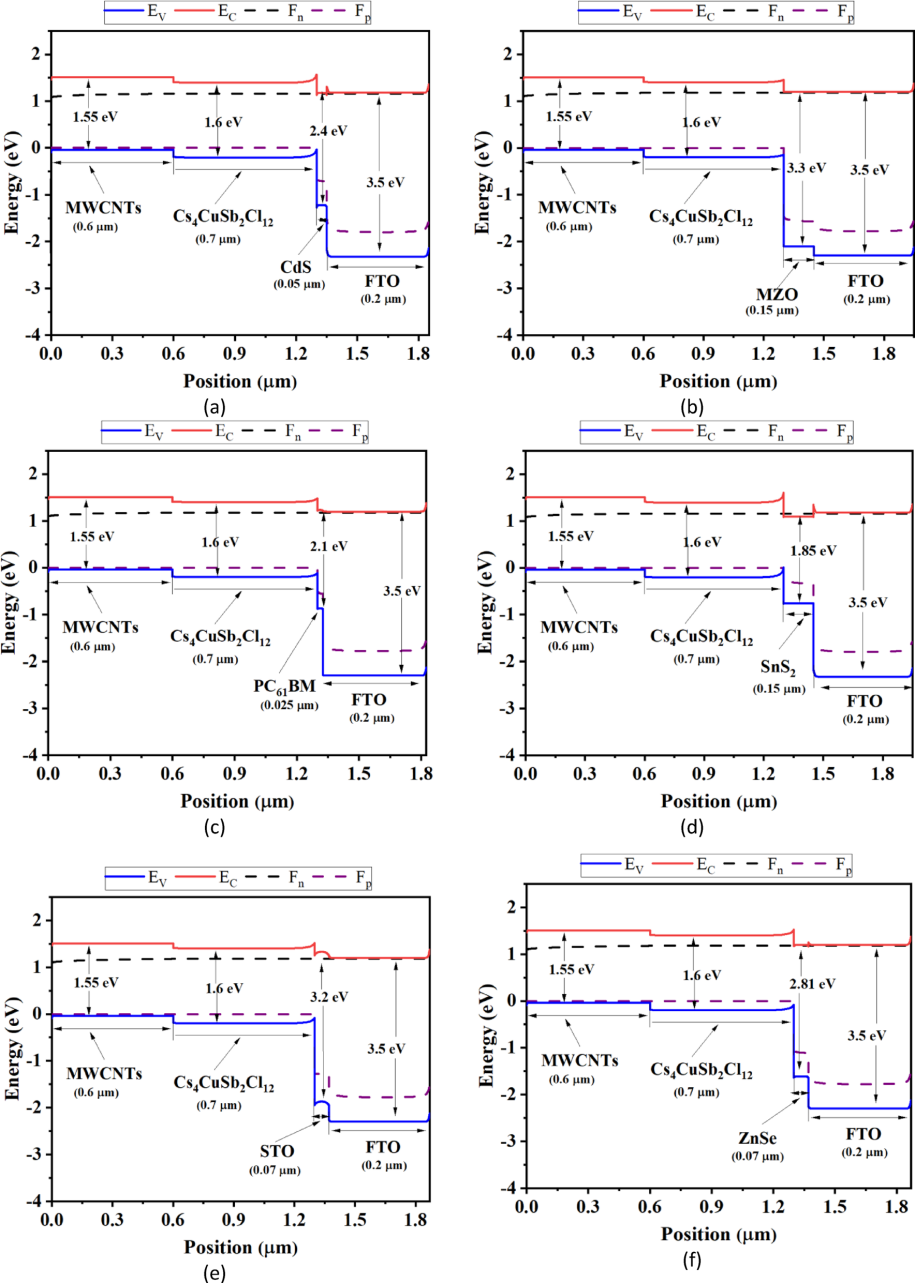
Energy band alignment of the studied PSC device structures based on various ETL materials with MWCNTs as the HTL.

Figure 2 represents the energy band diagrams for the studied PSC structures. Proper energy band alignment between layers is essential for efficient charge carrier transport^1,40^. The conduction band offset (CBO) and valence band offset (VBO) describe the energy difference between the conduction bands and valence bands, respectively, of two adjacent materials. These offsets are primarily influenced by the differences in electron affinity (for CBO) and ionization energy (for VBO). The electron transport layer (ETL) does not absorb photon energy; rather, its primary role is to facilitate the extraction and transport of photogenerated electrons from the absorber layer to the electrode. For efficient electron transport, a small or near-zero CBO at the ETL/absorber interface is preferred. If the conduction band edge of the absorber lies below that of the ETL, a spike-shaped CBO is formed, which may hinder electron extraction. Conversely, if the absorber’s conduction band is above the ETL’s, a cliff-shaped CBO occurs, which can promote recombination. Therefore, optimizing the CBO is critical for enhancing device performance^40,43^.

### Device optimization

#### HTL optimization

**Fig. 3:**
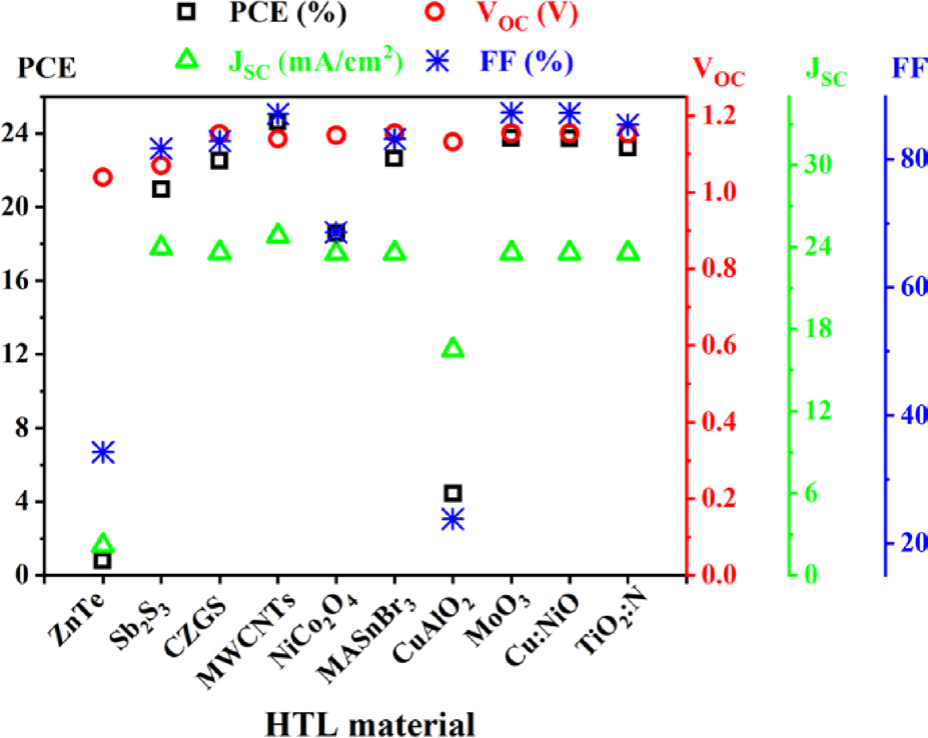
Comparison of performances of HTL materials on PSC parameters.

At the beginning of the work, the most suitable HTL layer for the Cs_4_CuSb_2_Cl_12_ absorber-based PSCs was selected. Among the studied HTLs ZnTe and CuAlO_2_ showed the poorest PCE of 0.77% and 4.44% (Fig. 3). Among other HTL materials Sb_2_S_3_, CZGS, NiCo_2_O_4_, MASnBr_3_, MoO_3_, Cu: NiO, and TiO_2_:N when used as the HTL layer showed 20.95%, 22.52%, 18.57%, 22.63%, 23.74%, 23.72%, and 23.22% PCE respectively in the Cs_4_CuSb_2_Cl_12_-based PSC structures. MWCNTs showed the best performance showing a 24.65% PCE in PSC structure when applied as an HTL layer. Also, V_oc_ of 1.14 V, J_sc_ of 24.82 mA/cm^2^, and FF of 87.07% were achieved for the device structure (Fig. 3). Here, the PSC structure with MWCNTs HTL showed 0.91% higher PCE than the second-best Cu: NiO HTL material. Considering overall performances, MWCNTs were chosen as the HTL material for further investigation.

#### Thickness optimization of absorber and ETL

**Fig. 4:**
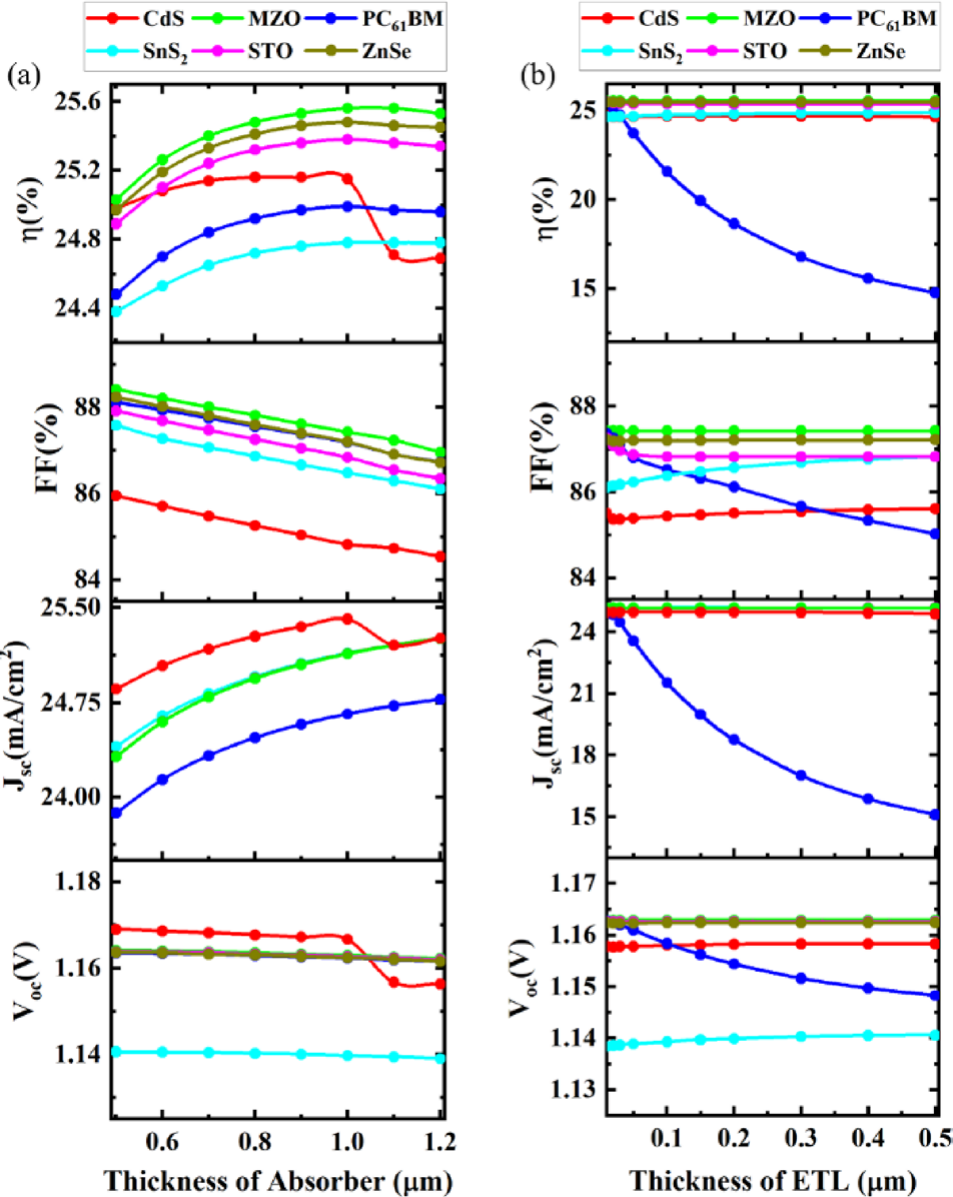
Effect of (**a**) thickness variation of absorber, and (**b**) thickness variation of ETL on device performance (PCE, FF, *V*_OC_, and *J*_SC_) .

To optimize the PSCs structure at first the impact of varying thickness of absorber material on six different device structures was studied. Figure 4(a) shows the effect on performances when absorber thickness was changed from 0.5 μm to 1.20 μm. As the thickness of absorber material increases PSCs can capture more photons which leads to more electron-hole pairs resulting in converting absorbed energy from photons to electricity. From Fig. 4(a), it is evident that the absorber showed the best PCE with the MZO ETL combination, reaching a PCE of 25.56% at 1 μm. Similar to PCE, Jsc increases until gets saturated when absorber thickness is enhanced. For tall the combinations J_sc_ increased as the thickness was increased. In all the combinations a stable FF was observed. For V_oc_, except for the combination with CdS structure, all others remained unaffected by the alteration of the thickness of the absorber. In the CdS ETL combination structure, a small slump of V_oc_ was observed at an absorber thickness of 0.8 μm and then it became saturated. Notably, the CdS-based structure exhibits a slight decrease in efficiency when the absorber thickness reaches 1.0 μm, which may be due to increased parasitic absorption in the CdS layer and enhanced recombination losses at the CdS/Cs_4_CuSb_2_Cl_12_ interface. This contrasts with other ETLs such as MZO and ZnSe, which maintain or improve performance due to their wider bandgaps and better optical transparency^64,65^.

Having an optimum thickness for the ETL layer helps to have minimal effect of recombination and provide effective performance. The best PCE for the structure in obtained for the different configurations when CdS, MZO, PC_61_BM, SnS_2_, STO, and ZnTe had a thickness of 0.15 μm, 0.15 μm, 0.02 μm, 0.15 μm, 0.03 μm and 0.05 μm thickness respectively. For the ETL layer at 0.15 μm, the highest PCE of 25.56% was achieved (Fig. 4(b)). For all parameters of FF, J_sc_, and V_oc_ all the ETL device structures showed relatively stable performances over the variation of ETL layers from 0.01 μm to 0.5 μm thickness except the PC_61_BM ETL combination showed degrading when thickness was increased. Therefore, a minimum of 0.02 μm was chosen as the optimum thickness for PC_61_BM ETL material. Figure 4(b) shows that performance variation with ETL thickness depends on material properties. Inorganic ETLs (e.g., MZO, STO, ZnSe) maintain stable performance due to high electron mobility and favorable energy alignment. In contrast, PC_61_BM shows a marked decline with increasing thickness, mainly due to its lower electron mobility and higher series resistance, which increase recombination and reduce charge collection efficiency^59^.

#### Effect of variation of doping density and defect density in the absorber

**Fig. 5:**
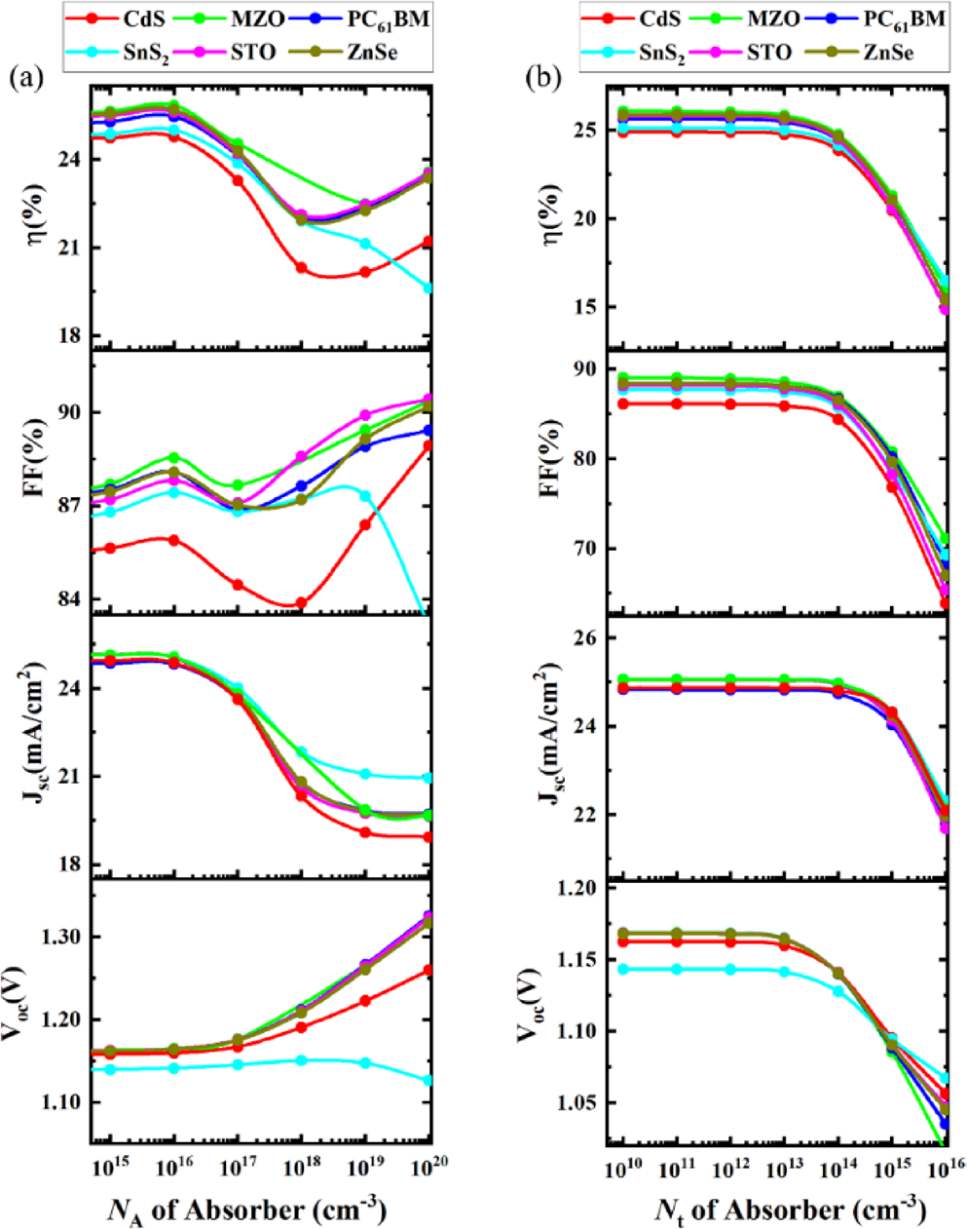
Significance of variation of absorber (**a**) acceptor density, and (**b**) defect density on device performances.

An increase in acceptor density helps the material’s ability to move holes more effectively and thus increases conductivity. However, enhancing acceptor density also leads to an increase in defects with a higher density which leads to deterioration of the overall PCE of the photovoltaic device. Figure 5(a) shows the effect on overall performances when the acceptor density of the absorber was studied between 10^15^ cm^− 3^ to 10^20^ cm^− 3^. For all the PSC combinations 10^16^ cm^− 3^ had the best PCE. After reaching an acceptor density of 10^16^ cm^− 3^ of absorber for all the structures PCE performance degraded. Among all the studied ETLs, the SnS_2_-based structure showed a comparatively sharper decrease in PCE beyond the optimum acceptor density of 10¹⁶ cm⁻³. This behavior is likely due to increased interfacial recombination resulting from the energy level mismatch and potential interface traps at the SnS₂/Cs_4_CuSb_2_Cl_12_ interface, which becomes more pronounced at higher doping concentrations^59,81,82^. Overall, the MZO ETL combination for absorbers with the highest PCE of 25.84% was achieved. Similarly to PCE, after reaching an optimum acceptor density for the absorber they had a fall in J_sc_ value since the value gets hampered by the presence of higher defect density due to the presence of a higher acceptor density.

Figure 5(b) represents the effect of the total defect density of absorber material. A higher defect density in PSC absorbers leads to trapping charge carriers. As a result, it can increase recombination and have a negative impact on an electric field. So, both V_oc_ and FF get affected directly which also leads to the deterioration of PCE performance. In Fig. 5(b), the defect density of the absorber was varied from 10^10^ cm^− 3^ to 10^16^ cm^− 3^. Until 10^13^ cm^− 3^ PCE, V_oc_, J_sc_, and FF had stable results. After reaching the threshold defect density, impaired material properties started to lead to a quick fall in performance in all device structure combinations. Therefore, 10^13^ cm^− 3^ can be considered the highest tolerable defect density of the absorber.

#### Effect of the variation of donor density of ETL and acceptor density of HTL

**Fig. 6:**
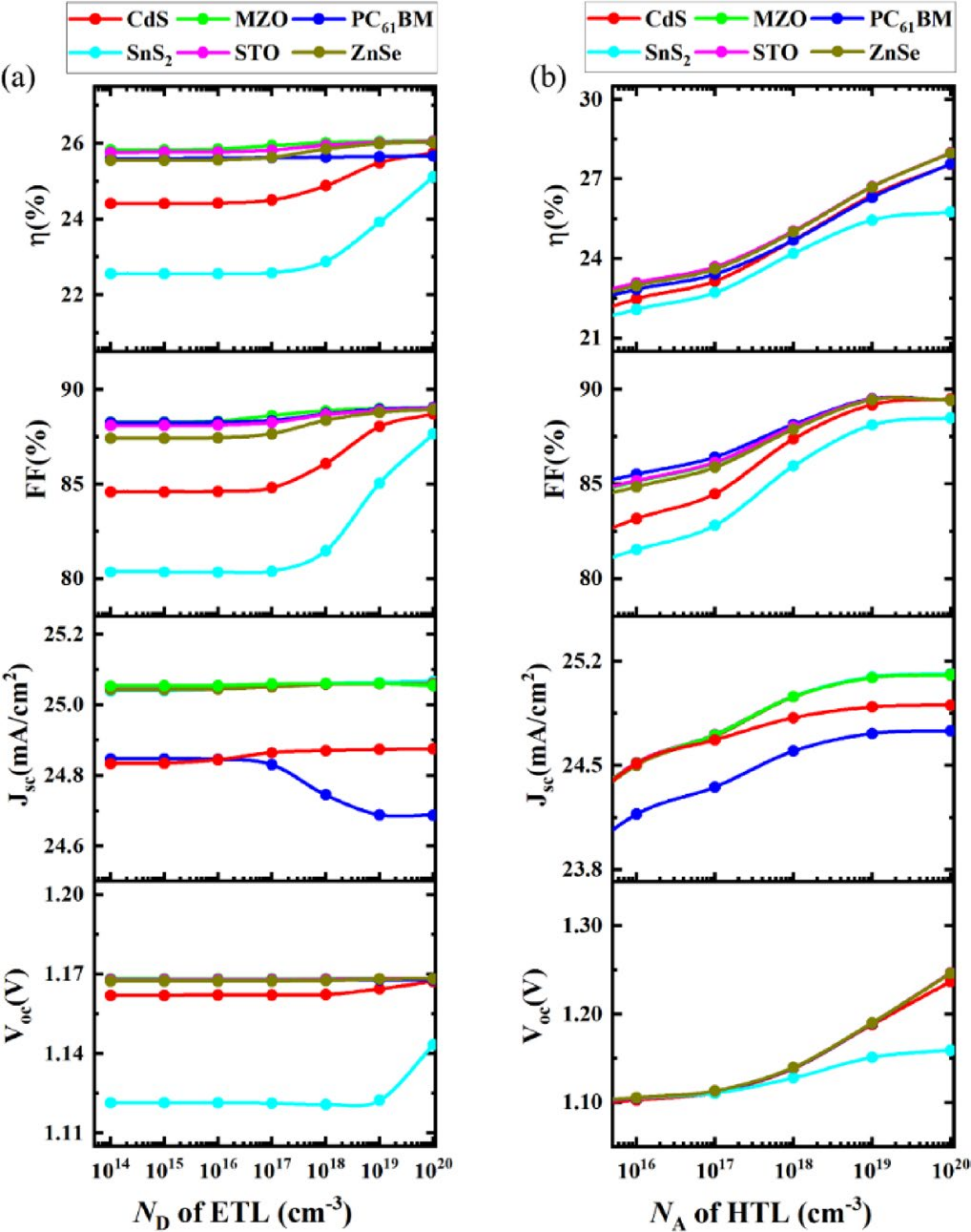
Effect of (**a**) donor density of ETL, (**b**) acceptor density variation of HTL on device performances.

Figure 6(a) shows the effect of changing the donor density of ETL from 10^14^ cm^− 3^ to 10^20^ cm^− 3^. When SnS_2_ and CdS ETLs reached donor density at 10^17^, a sharp rise in PCE and FF was witnessed in their structures. PCE and FF remained steady for the change in other ETLs. J_sc_ was stable in PSCs when donor density was varied in MZO, SnS_2,_ ZnSe, and STO ETLs. However, a minor increase was found in the CdS ETL layer’s device, and a decrease was witnessed in the combination of the PC_61_BM layer’s device. V_oc_ was increased when the donor density of SnS_2_ and CdS ETLs were enhanced from 10^19^ cm^− 3^ to 10^20^ cm^− 3^ in the PSCs. The change had no visible impact on other device structures^67^.

Figure 6(b) represents the impact of acceptor density variation on PSCs. Having a higher acceptor density in HTLs helps to have a strong electron field presence at the interface of the absorber and HTL. It helps to minimize recombination and separate electron-hole pairs more effectively. Therefore, it affects PSC by improving performance in general. From Fig. 6(b), a similar expected performance can be seen. Acceptor density for ETLs varied from 10^15^ to 10^20^ cm^− 3^ where a surge in PCE was observed. Device structures having CdS, MZO, PC_61_BM, SnS_2_, STO, and ZnTe HTLs showed improved PCE from 21,48% to 27,56%, 22.28–27.99%, 22.08–27.57%, 21.28–25.75%, 22.29–27.29% and 22.18–27.98% respectively.

### Effect of various parameters on PV performance

#### Effect of series resistance, shunt resistance, and temperature on device

**Fig. 7:**
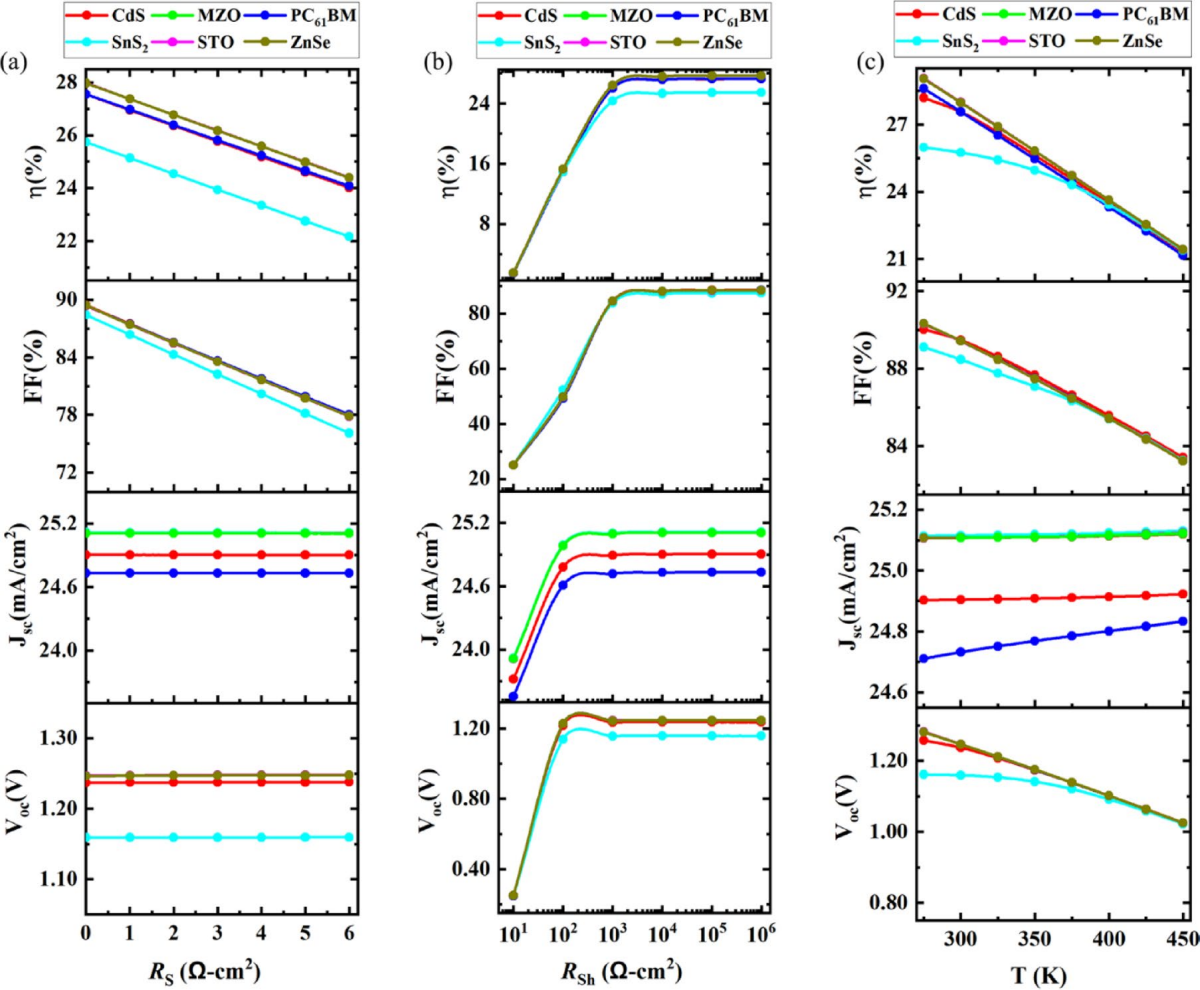
Effect of variation of (**a**) series resistance, (**b**) shunt resistance, and (**c**) temperature on *V*_OC_ (V), *J*_SC_ (mA cm^− 2^), FF (%) and η (%).

The combined resistance of metal layers and semiconductor layers is known as series resistance (R_s_). High series resistance causes hampers in carriers’ mobility in PSCs that result in the degrading performance of PCE and FF. Figure 7(a) shows series resistance was studied between 0 Ω-cm^2^ to 6 Ω-cm^2^. For all the device structures PCE and FF decreased linearly. Whereas J_sc_ and V_oc_ maintained an unchanged performance over the variation of series resistance. This behavior can be explained by the role of R_s_ in the solar cell operation. Series resistance primarily impacts the current flow in the external circuit and causes power losses during load conditions, which significantly affects FF and PCE^59^. However, J_sc_ is determined under short-circuit conditions (zero voltage), where R_s_ has minimal influence, and V_oc_ is derived from the intrinsic properties of the absorber and junction, which are not directly altered by R_s_. Therefore, R_s_ has a negligible effect on these two parameters within the studied range. Overall, the decline in PCE was not more than 4% in all of the PSC combinations and the device structures showed reliable performance when a higher series resistance was considered.

Figure 7(b) represents the effect of shunt resistance (R_sh_) on our PSC devices. The shunt is the resistance in the junction of PSC that provides an alternative path for the current. Lower shunt resistance causes a high loss of PCE whereas higher shunt resistance can help to bypass the intended path to the external circuit more effectively, hence showing improved overall performances. Here, shunt resistance was studied from 10 Ω-cm^26^ to 10 Ω-cm^2^. In all combinations, PCE and FF increased sharply from 10 Ω-cm^23^ to 10 Ω-cm^2^ and then both PCE and FF became stable. However, beyond a certain threshold (10³ Ω·cm²), the shunt resistance becomes sufficiently high that leakage currents are effectively negligible. At this stage, further increases in Rsh do not significantly impact the current pathways or performance of the device, leading to a saturation in the values of PCE, FF, J_sc_, and V_oc_. Hence, all PV parameters remain unchanged for R_sh_ values greater than 10³ Ω·cm². This plateau indicates the device has reached an optimal regime where shunt resistance no longer limits performance^81^. The STO and MZO ETL combination PSCs showed the highest PCE of 27.69% at 10^6^ Ω-cm^2^. J_sc_ and V_oc_ achieved became saturated at 10^12^ Ω-cm^2^ in all PSC structures.

Higher temperature has an inverse relationship with PCE and FF. It increases resistance and leads to more recombination, which reduces the overall performance of the PSC. So, PCE and FF deteriorate when at higher temperatures. Figure 7**(c)** shows the effect of temperature variations from 275 K to 450 K. At 275 K temperature the highest PCE was observed in the structure with an STO ETL layer of 29.06% followed by the structure having a ZnSe ETL layer of 29.04%. Among all the PSCs, the combination with SnS_2_ showed the best resilience over the enhancement of temperature. Its PCE at 275 K was 25.98% which dropped to 21.43% at 450 K, meaning a decrease of 4.55%which is smaller than the decrease observed in PSCs with MZO, STO, CdS, and PC_61_BM ETLs, where the PCE dropped by more than 7%. In FF and V_oc_ output results a comparable change is observed to PCE. A small linear increment in J_sc_ was found when the temperature was increased in the PSC structures. An increase in temperature provides more thermal energy and enhances the electron-hole generation rate, hence increasing J_sc_ in the output^59,81,82^.

#### Effect of variation of absorber thickness with absorber defect density

**Fig. 8:**
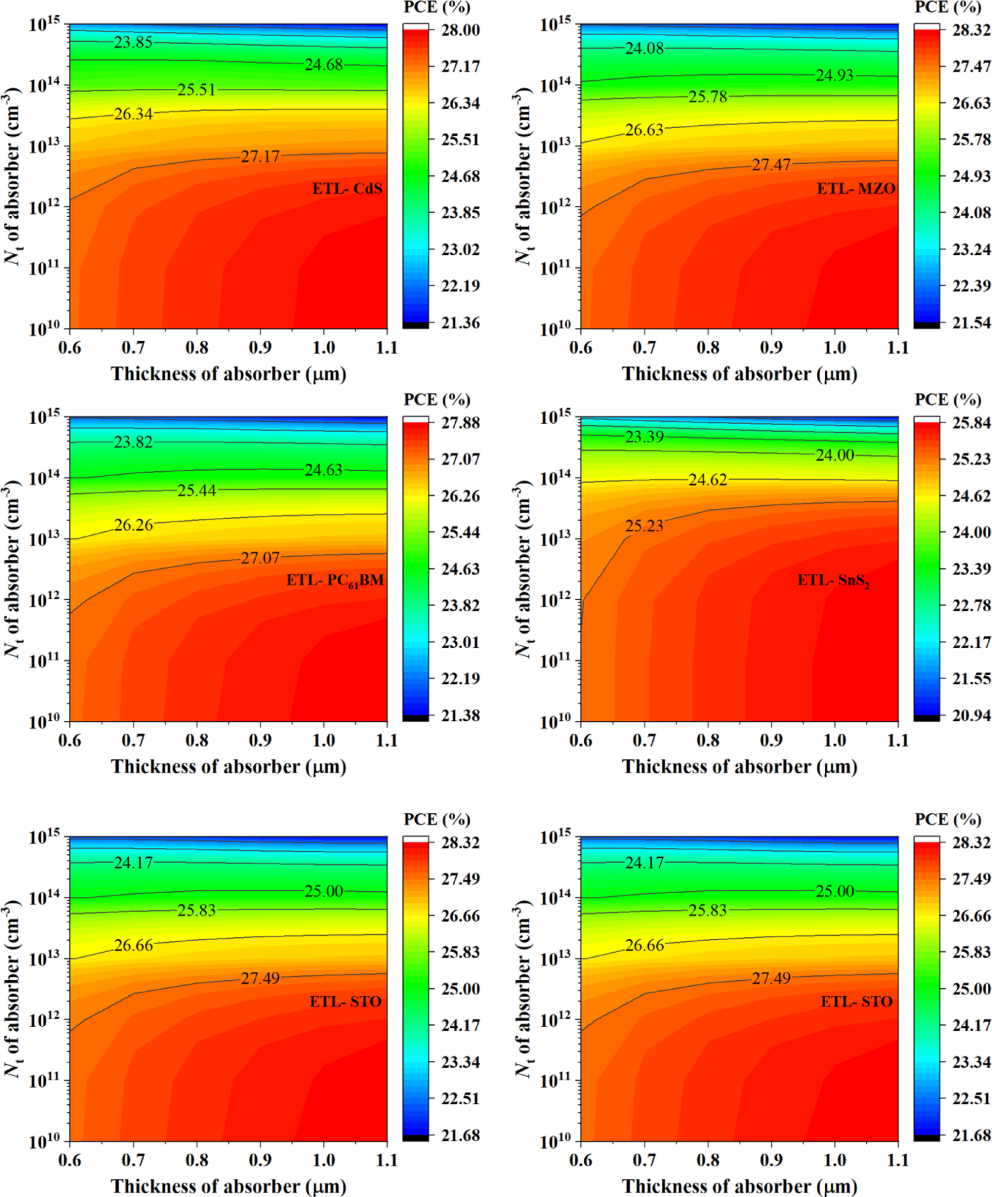
Contour mapping of PCE photovoltaic parameter with respect to thickness of absorber, and total density of absorber in device structures based on various ETL materials.

Figure 8 represents the contour mapping of the impact of variation of the absorber layer thickness and with the change of total defect density of absorber N_t_. The presence of N_t_ in heterojunction devices negatively impacts performances by curbing the mobility of carriers. For the studied devices the best PCE performances were observed when the thickness of the absorber layer was between 950 nm and 1100 nm. From 10^10^ to 10^12^ cm^− 3^ the absorber showed good stability in performances. At a defect density of 10^10^ cm^− 3^, a noticeable increase in V_OC_ was observed due to the minimal recombination losses in the simulation, which does not account for interfacial defects. This idealized scenario can temporarily elevate V_OC_ values beyond typical limits like the Shockley–Queisser threshold, highlighting the impact of ultra-low defect densities in enhancing carrier lifetimes and quasi-Fermi level splitting^40,43,83^.

For the studied six PSCs its stable performance in PCE was observed from 10^10^ to 10^12^ cm^− 3^. In the CdS ETL-based device, the PCE of 27.99% was observed when the studied thickness of the absorber was 1100 nm and N_t_ 10^10^ cm^− 3^. In the MZO-based ETL structure, a maximum of 28.31% PCE was noted at an absorber thickness and N_t_ of 1100 nm and 10^10^ cm^− 3^. For, the same optimum conditions other ETL materials-based PSCs showed their best PCE. When PC_61_BM, SnS_2_, STO, and, ZnSe were used as the ETL layers the highest PCE was noted at 27.88%, 25.84%, 28.31%, and 28.3% respectively.

#### Effect of the variation of acceptor density of absorber with defect density of absorber

**Fig. 9:**
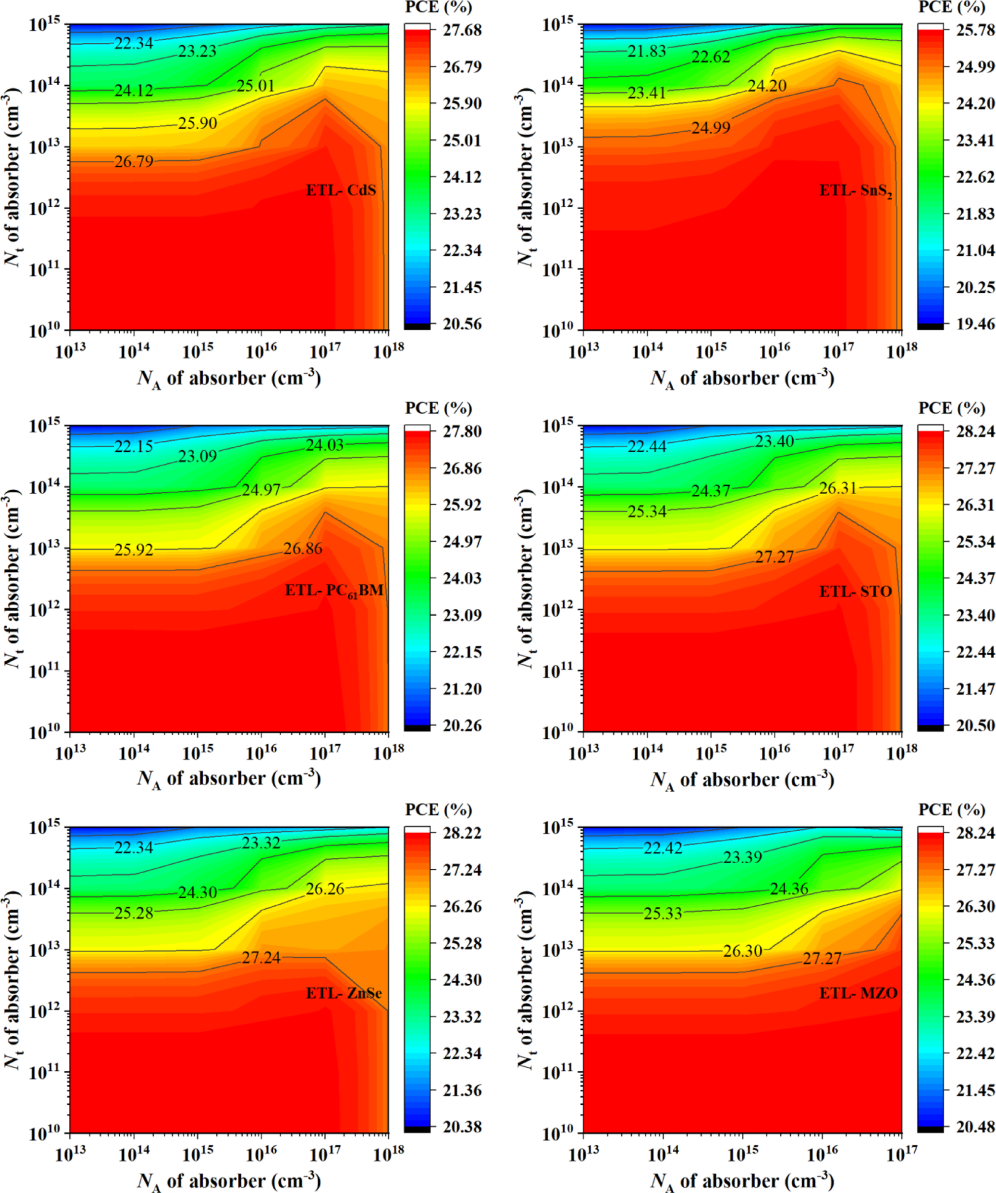
Contour mapping of PCE photovoltaic parameter with respect to acceptor density and total defect density variation on various ETL materials based PSC devices.

Figure 9 shows the contour mapping of the variation of acceptor density and defect density variation of absorber where acceptor density was varied from 10^13^ to 10^18^ cm^− 3^ and defect density was changed from 10^10^ to 10^15^ cm^− 3^ for the studied PSC devices. Higher defect density in semiconductor materials of PSCs is unwanted since it leads to the degradation of performances. However, defects in materials are found so the impact of it has to be considered while designing PSCs. Materials that show high stability at comparatively higher defect density can be considered ideal for practical implementation. For the variation of acceptor density and defect density, Fig. 9 shows all the device structures maintained high PCE at 10^13^ cm^− 3^. When choosing the ideal acceptor density, an optimum density is needed. Low acceptor density in absorber lacks effectiveness and a high density can increase recombination leading to an unoptimized PCE performance. From Fig. 9, the optimum condition for PCE was found at the acceptor density of the absorber of 10^17^ cm^− 3^. Among the studied PSCs for both MZO and PTO ETL-based device structures the highest PCE of 28.23% was found when the acceptor density of the absorber was 10^13^ cm^− 3^ and the defect density of the absorber was 10^10^ cm^− 3^. It is important to note that the optimum acceptor density of 10^13^ cm^− 3^ reported here corresponds to a condition of ultra-low defect density (10^10^ cm^− 3^). This contrasts with the single-variable study in Sect. 3.2.3, where an acceptor density of 10^16^ cm^− 3^ was found optimal under a fixed defect density. This highlights the importance of considering combined parameter interactions when optimizing device performance.

#### Effect of carrier generation and recombination rates

**Fig. 10:**
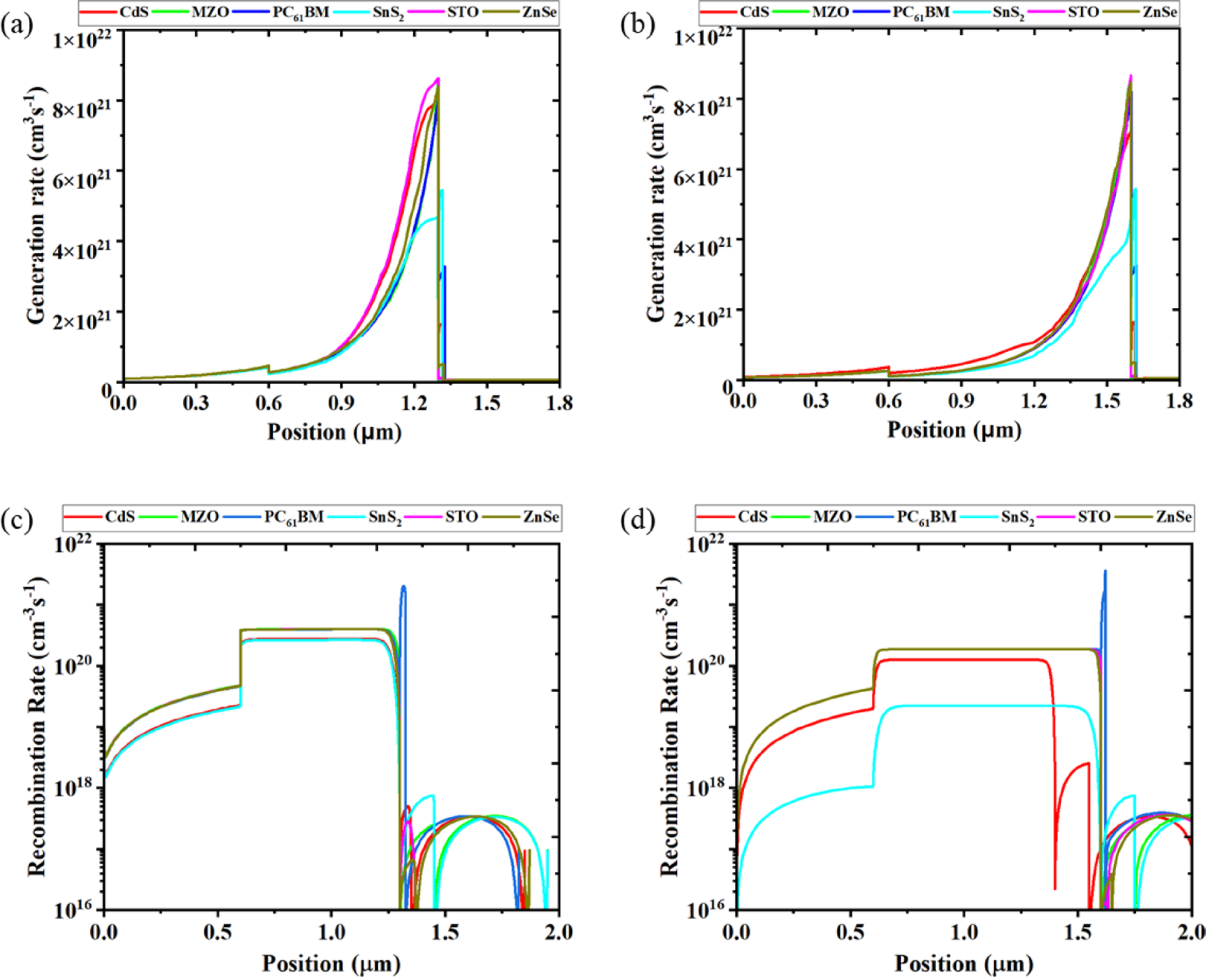
Carrier generation rate (**a**) before optimization (**b**) after optimization in the device structures. Carrier recombination rate (**c**) before optimization, (**d**) after optimization in the device structures.

Figure 10 illustrates the spatial distribution of photogenerated carriers within the studied PSCs. Carrier generation, the process of creating electron-hole pairs through photon absorption, is primarily concentrated in the absorber layer, as shown in Fig. 10(a). However, through structural optimization, the generation profile can shift toward the ETL side, as seen in Fig. 10(b), resulting in a more distributed and efficient generation of charge carriers throughout the device. This shift is achieved by optimizing the ETL properties such as thickness, doping concentration, and energy level alignment. A well-optimized ETL enhances the absorption of incident light near the ETL/absorber interface and facilitates efficient charge extraction^40,43,59,64^. These modifications reduce carrier recombination, improve band alignment for smoother electron transport, and maintain a strong internal electric field to support carrier separation and collection. As a result, devices show increased carrier lifetimes and improved performance metrics, particularly in J_sc_ and FF. Therefore, the optimization process involves tailoring both the optical and electronic environments of the ETL and absorber layers. By controlling where carriers are generated and ensuring their efficient extraction, overall device efficiency is enhanced^59,67,81^.

Figure 10(c) and 10(d) present the carrier recombination profiles across the device structure before and after optimization, respectively. Recombination, the loss mechanism where photogenerated electrons and holes annihilate without contributing to current, is a critical factor in determining the photovoltaic performance^1,40,43^. In the unoptimized structure (Fig. 10(c)), elevated recombination rates are observed, particularly near the absorber interfaces, indicating inefficient charge extraction and higher losses. After optimization (Fig. 10(d)), a notable suppression of recombination is achieved throughout the active layers, especially near the ETL/absorber and absorber/HTL interfaces. This reduction is attributed to improved energy level alignment, enhanced carrier mobility, and reduced defect densities introduced by tuning the ETL parameters. Lower recombination not only leads to higher carrier lifetimes but also contributes to increased open-circuit voltage (V_oc_) and fill factor (FF). Thus, minimizing recombination alongside enhancing generation ensures that more carriers contribute to the photocurrent, enabling a more realistic and efficient device performance^40,43,59,64^.

### Analysis of J–V characteristics and quantum efficiency of PSC structures

**Fig. 11:**
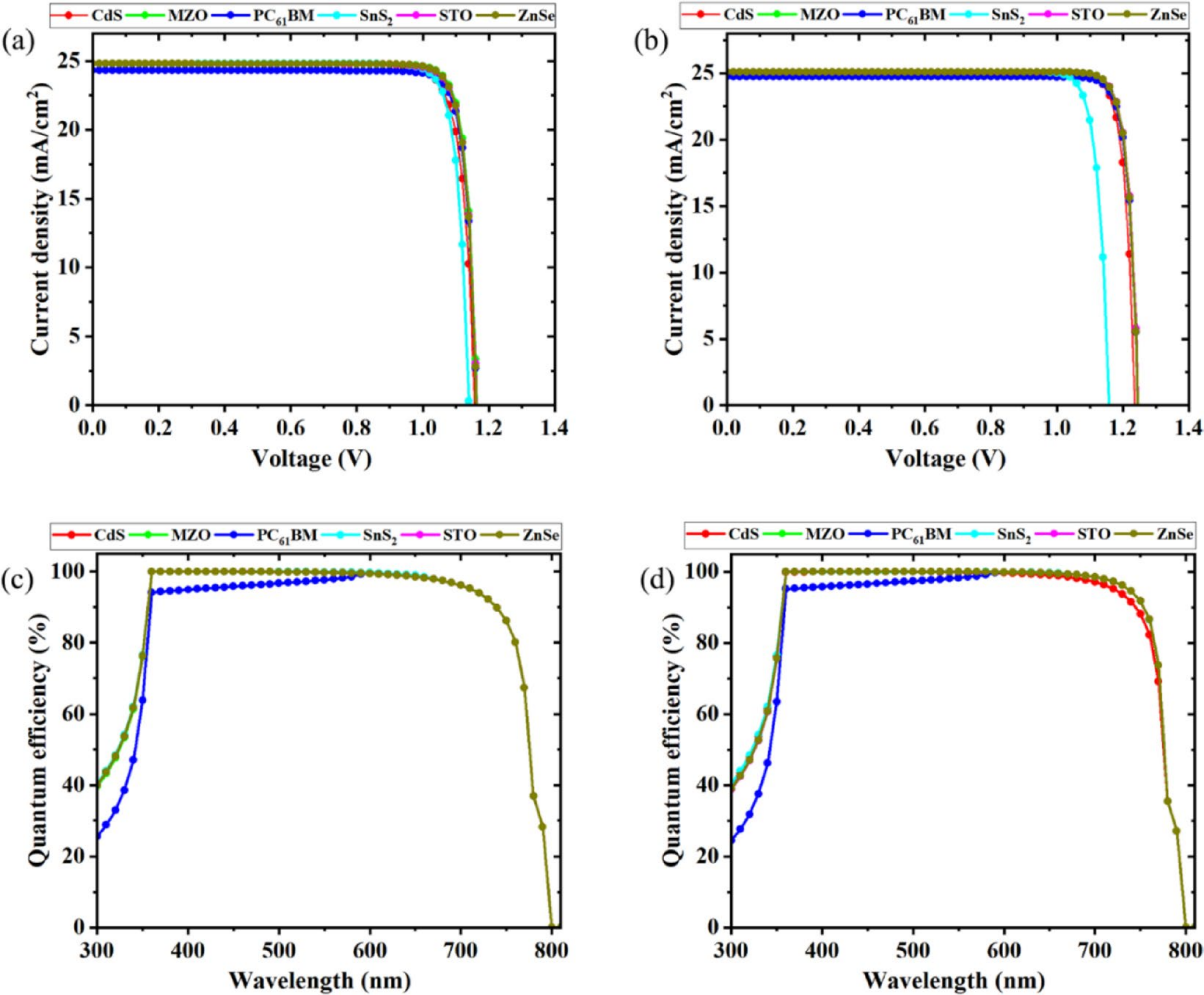
(**a**) J-V curve before optimization, (**b**) J-V curve after optimization, (**c**) quantum efficiency before optimization, and (**d**) quantum efficiency after optimization in the device structures.

Figures 11(a) and (b) illustrate the current density and open-circuit voltage (V_oc_) of the PSCs before and after optimization. The current density remains largely unchanged, indicating consistent carrier collection across all configurations. In contrast, Voc shows a noticeable increase after optimization, suggesting a reduction in non-radiative recombination and better energy level alignment at interfaces. Since the FF is directly influenced by V_oc_, this enhancement contributes to an overall improvement in PCE^59,67,81^.

Figures 11(c) and (d) present the external quantum efficiency (EQE) spectra before and after optimization. EQE measures the efficiency with which incident photons are converted into charge carriers at each wavelength. Across all devices, a peak EQE of nearly 100% is observed around 360 nm, particularly for CdS, MZO, SnS₂, STO, and ZnSe-based ETLs. After optimization, a slight increase in EQE is noted over a broad spectral range (300–800 nm), confirming improved carrier generation and collection^40,43,59,64^.

Although the spectral response range does not expand significantly post-optimization, the observed enhancements in V_oc_ and EQE confirm better device operation due to reduced losses and improved interfacial quality. This indicates that the optimization process mainly enhanced charge transport and reduced recombination rather than altering the optical absorption characteristics of the absorber layer^40,43,59,64^. Absorber thickness and optical modeling were not varied in this specific analysis, and their effect on light harvesting may be considered in future work.

### Comparison of results with previous works

Table 4 presents a comparison of the recent research works^76–78,99^ on the Cs_4_CuSb_2_Cl_12_ absorber PSC devices. Among the studies, only one work^78^ shows over 25% PCE after device optimizations. To close the gap of existing knowledge on Cs_4_CuSb_2_Cl_12−_absorber-based PSCs, in this work, an extensive study was carried out on charge transport layers. Over 27% PCE was obtained under the optimized condition for the six different ETL materials.

**Table 4:** Comparison of lead-free Cs_4_CuSb_2_Cl_12_-based PSCs.

Device structure	PCE (%)	Reference
FTO/IGZO/Cs_4_CuSb_2_Cl_12_/CuO/Au	17.39%	^99^
FTO/TiO_2_/Cs_4_CuSb_2_Cl_12_ nanocrystals/Cu_2_O/Au	23.07%	^76^
FTO/WS_2_/Cs_4_CuSb_2_Cl_12_/CuSbS_2_/Ni	23.10%	^77^
FTO/SnO_2_/Cs_4_CuSb_2_Cl_12_/CuSCN/counter electrode (Au)	29.71%	^78^
FTO/SnO_2_/Cs_4_CuSb_2_Cl_12_/counter electrode (Pt) (HTL-free)	29.69%	^78^
Al/FTO/STO/Cs_4_CuSb_2_Cl_12_/MWCNTs/Au	28.23%	This work
Al/FTO/MZO/Cs_4_CuSb_2_Cl_12_/MWCNTs/Au	28.23%	This work

Among the studied HTL materials, MWCNTs highlighted superior performances to other studied materials so it was chosen as the preferred HTL material here. Both MZO and STO ETLs in the device structures showed 28.23% PCE. Among these materials, MZO consists of elements that are cheap and less toxic. However, it faces adversity to the exposure of moisture therefore, water-restrained additive methods in MZO are used for better durability^100^. STO is comparatively more expensive and detrimental to the environment. STO is not susceptible to moisture and has excellent chemical and thermal stability making it a great choice to be deemed for longevity^101,102^. Overall, the (Al/FTO/MZO/Cs_4_CuSb_2_Cl_12_/MWCNTs/Au) device structure is appropriate to maintain cost-effectiveness and to ensure less environmental damage. (Al/FTO/STO/Cs_4_CuSb_2_Cl_12_/MWCNTs/Au) device structure can be considered if getting the best long-term durability in PSC performances is the primary priority.

## Conclusion and future outlook

In this work, among 10 studied HTL materials, MWCNTs were selected as the best-performing HTL layer in the Cs_4_CuSb_2_Cl_12_ PSC device structure for further exploration. Al/FTO/STO/Cs_4_CuSb_2_Cl_12_/MWCNTs/Au and Al/FTO/MZO/Cs_4_CuSb_2_Cl_12_/MWCNTs/Au device structures demonstrated the best performances, showing over 28% PCE after optimization. Increasing the thickness of the absorber layer for the studied structures showed enhancement in performance and showed the best PCE when the thickness was 1 μm. PSC structure having CdS ETL showed a big drop off in performance compared to the other devices’ structure when the thickness was increased further. On the other side, the performances of PSC structures were affected slightly with the increase of ETL thickness except the PC_61_BM affected the PSC structure significantly when the thickness was increased from 0.02 μm.

Further optimization of absorber properties revealed that The acceptor density of 10^16^ cm^− 3^ of absorber showed the best performance on the studied devices. The defect density of the acceptor was stable until 10^14^ cm^− 3^. After that, increasing further total defect density sharply damaged the PSC devices’ performances. Donor densities of ETLs and acceptor densities of HTL were examined. As ETL materials MZO, and STO had the best donor density in the Cs_4_CuSb_2_Cl_12_ -PSC device at 10^18^ cm^− 3^ and 10^16^ cm^− 3^ respectively. Changing temperature exhibited linear degradation in all the studied devices. Overall, the examined device structures demonstrated excellent PCE at 300 K and over 20% PCE at an extreme 450 K temperature. A similar effect was noticed when series resistance was enhanced. Shunt resistance showed the optimum performance from 1000 Ω-cm^2^and showed saturated performance when the resistance was enhanced further. These findings emphasize the crucial role of device physics parameters in determining overall stability and efficiency, highlighting the value of multi-parameter optimization.

Post-optimization, J–V and quantum efficiency (QE) curves confirmed significant performance improvements in the best-performing structures. These included an increase in open-circuit voltage and enhanced quantum efficiency across the visible range. Such improvements validated the effectiveness of the chosen transport layers and structural modifications in boosting device performance. This indicates that a combination of proper energy alignment, material selection, and structural optimization can significantly improve charge extraction and light absorption, leading to high device performance under various conditions.

In future works, organic materials can be widely studied as charge transport layers for the Cs_4_CuSb_2_Cl_12_ PSCs. Besides, device modifications such as the effect of the back-surface field layer, and the effect of double HTLs can be considered. Studied MZO could be considered the best performing ETL if we consider cost and toxicity issues, though STO could show better durability than MZO. The findings of these studies can be further researched by working experimentally by following the framework of this work. Overall, this study provides a comprehensive framework for developing efficient, lead-free PSC devices and serves as a valuable reference for experimental validation and future commercialization efforts.

## Data Availability

The raw/processed data required to reproduce these findings cannot be shared at this time as the data also forms part of an ongoing study and are available from the corresponding author on reasonable request.
